# Before we knew to ask: guidance families need in cancer care

**DOI:** 10.1093/oncolo/oyag305

**Published:** 2026-07-30

**Authors:** Joseph A Bauer

**Affiliations:** Bauer Research Foundation, Inc. CCC (Cancer Care Concierge and Chronic Care Concierge), Akron, OH 44312-5272, United States

When my father was diagnosed with glioblastoma, I believed that if nothing else, my family would understand the road ahead.

I had spent years in cancer research and had served on the professional staff of a major cancer center. I knew the institution, the language of oncology, and the seriousness of the diagnosis. I did not imagine that this knowledge would soften the blow. But I did assume it would help us see the process clearly. I thought we would understand how the pieces of care fit together, what questions were likely to arise, and what resources might become important as the illness progressed.

Instead, we found ourselves where so many families do: overwhelmed, reactive, and trying to assemble a coherent picture from separate conversations.

My father’s care unfolded through surgery, radiation oncology, and medical oncology, each focused on its own part of the problem. He underwent a laser interstitial thermal therapy procedure. Radiation followed. Temozolomide was added, as it is for many patients with glioblastoma. The issue was not that these decisions were unreasonable or that the clinicians were indifferent. The issue was that no one helped us understand the larger picture in a way we could carry with us. We moved from one step to the next without a clear sense of what might reasonably come next, what kinds of support might become necessary, or who would help us navigate the whole.

That omission shaped our experience.

I do not mean that every turn in a serious illness can or should be predicted. Oncologists do not have a crystal ball. They live with the hope that the next patient may do better than expected. A one-day-at-a-time approach can also protect patients and families from feeling crushed by possibilities that may never come to pass. I understand that, and I do not write this to ask for certainty.

What I am describing is something more practical: the need for guidance about what may lie ahead, what support exists, and when those supports should enter the conversation.

When my father’s speech began to deteriorate, our fear rose quickly. We called and asked what could be done. Only then did we learn that speech therapy, occupational therapy, and physical therapy could be arranged in the home. Those services were made available promptly once we asked. I was grateful for that. But I was also left wondering why no one had said earlier, “If this becomes an issue, these are the resources we can mobilize.” We did not need a prediction that decline would happen on a specific timetable. We needed to know, before we were frightened and scrambling, that help existed.

The same was true of emotional support. My father was depressed, as many patients with serious cancer understandably are. Yet psycho-oncology was never meaningfully introduced to us as a resource. No one framed emotional suffering as an expected part of the experience and explained how that burden might be addressed. We learned about such support only later, after the need had already become obvious to us at home.

The same pattern returned near the end of his illness. When my father became bedridden, we began asking about hospice. Again, the answer was yes, that support was available. Again, I wondered why it had not been discussed earlier, not as an immediate next step, but as part of the larger landscape families may eventually have to face. Hospice, palliative care, rehabilitation, counseling, and home services should not feel like things families discover only in crisis.

What stayed with me most was the realization that my family entered this experience with unusual advantages. I knew the institution. I knew the vocabulary. I knew enough to ask more informed questions than most families can reasonably be expected to ask. And still, we often felt behind. If we felt that way, what must this be like for families with no insider knowledge at all?

That question changed the way I think about quality in cancer care.

My father’s physicians were not uncaring, and the institution was not lacking in expertise. The problem was more subtle than that. Technical excellence and family understanding are not the same thing. A center can provide sophisticated treatment and still leave patients and families unsure who is coordinating care, what support exists, and when to seek it. In that setting, families become the default managers of a complicated process at the very moment they are least equipped to do so.

Clinical trials were part of that experience as well. No meaningful discussion took place about possible trial opportunities outside the treating institution. I later became aware of another option elsewhere, but by then the information no longer had the same value it might have had earlier. I understand that trial decisions are complicated and that not every patient is a candidate. My concern is narrower: families should come away with a clearer understanding of what was considered, what was not, and why.

To be sure, many cancer centers do have patient navigators, social workers, psycho-oncology programs, palliative care teams, and family-support services. The problem is not that these resources never exist. The problem is that they may not be introduced in a way that is timely, visible, and usable when the need first begins to emerge. Some are overextended. Some are embedded so deeply within institutional workflows that families may not realize they are available at all. There is a meaningful difference between a resource existing and a family actually experiencing it as part of care.

That experience stayed with me long after my father died. It shaped my conviction that patient navigation must be more than a service added after the fact. Families need care coordination, advocacy, practical resources, clear education, and support built into the journey from the beginning—not discovered one crisis at a time.

We speak often in oncology about innovation. Usually we mean a new drug, a new device, a new molecular target, or a new trial design. Those advances matter profoundly. But for many families, one of the most meaningful improvements would be simpler: clearer guidance about what may come next, what help exists, and when to ask for it.

My father did not need false reassurance. Our family did not expect certainty. What we needed was guidance before we knew to ask.

